# Massachusetts

**Published:** 1896-10

**Authors:** 


### CONTROL WORK.

*Massachusetts.* A herd of cattle at New Salem was recently reported as tuberculous. This herd had been brought from another territory, and it is said that the native cattle of that district enjoy almost complete immunity from tuberculosis.

A number of hogs are reported to have died at Hinsdale from tuberculosis.

